# Residential Place Attachment as an Adaptive Strategy for Coping With the Reduction of Spatial Abilities in Old Age

**DOI:** 10.3389/fpsyg.2019.00856

**Published:** 2019-04-24

**Authors:** Ferdinando Fornara, Amanda Elizabeth Lai, Marino Bonaiuto, Francesca Pazzaglia

**Affiliations:** ^1^Department of Education, Psychology, Philosophy, University of Cagliari, Cagliari, Italy; ^2^Interuniversity Research Center in Environmental Psychology (CIRPA), Rome, Italy; ^3^Center for Research in Psychology, Autonomous University of Lisbon, Lisbon, Portugal; ^4^Department of Social and Developmental Psychology, Sapienza University of Rome, Rome, Italy; ^5^Department of General Psychology, University of Padua, Padua, Italy

**Keywords:** place attachment, residential satisfaction, spatial self-efficacy, spatial anxiety, wayfinding, elderly population, adaptation strategy, docility hypothesis

## Abstract

This study intended to test whether attachment to one’s own residential place at neighborhood level could represent a coping response for the elderly (consistently with the “docility hypothesis;” Lawton, 1982), when dealing with the demands of unfamiliar environments, in order to balance their reduction of spatial abilities. Specifically, a sequential path was tested, in which neighborhood attachment was expected to play a buffer role between lowered spatial competence and neighborhood satisfaction. The participants (*N* = 264), senior citizens (over 65-year-old), responded to a questionnaire including the measures of spatial self-efficacy, spatial anxiety, attitude toward wayfinding, residential attachment and residential satisfaction. Results from the mediation analysis showed that a lower perceived spatial self-efficacy is associated to a higher spatial anxiety, and both promote a more negative attitude toward wayfinding tasks in non-familiar places. This leads to a higher attachment to one’s own neighborhood, which in turn predicts a higher residential satisfaction. Thus, the “closure” response of becoming more attached to their residential place may be an adaptive strategy of the elderly for compensating the Person-Environment (P-E) *mis-*fit (Lawton and Nahemow, 1973) when they feel unable (or less able) to cope with the demands of unfamiliar environments.

## Introduction

This contribution is focused on the role of residential place attachment as an adaptive coping strategy in contrasting the reduction of orientation abilities in non-familiar environments in older adults. The capacity to orientate oneself in the surrounding environment and finding the way to get to a destination is a crucial ability for human survival.

Orientation abilities have been typically measured by the use of objective environment tasks (e.g., spatial navigation, direction and distance estimation, and map drawing), and subjective measures, such as self-reported measures of sense of direction (SOD; e.g., Kozlowski and Bryant, 1977), spatial anxiety (Lawton, 1994), spatial efficacy (Mitolo et al., 2015), and spatial attitude scales (Meneghetti et al., 2014). Importantly, self-reported measures of environmental abilities (such as SOD) have been found to highly correlate to each other (Meneghetti et al., 2014; Mitolo et al., 2015) and to highly predict objective measures of these abilities (Hegarty et al., 2006; Pazzaglia et al., 2017).

It is widely acknowledged that spatial abilities decline during old age: older people are less able to perform wayfinding tasks than younger people (Wilkniss et al., 1997), they also have less orientation after reading a map and they are less able to estimate distance (Ellis and Young, 1990). Nevertheless, familiarity and experience of places can successfully play a buffer role among older adults, thus counterbalancing their competence gap. In fact, the elderly’s orientation in familiar environments (e.g., one’s own residential environment) was found to be similar to those of other ages, at home or at city level (Ohta and Kirasic, 1983). Surprisingly, there is lack of evidence in literature about the relationship between spatial competence patterns and residential place attachment.

Place attachment is a key construct developed in the environmental psychology domain for describing a relationship between people and places. Most definitions in literature have stressed the affective (e.g., see Riley, 1992; Hidalgo and Hernandez, 2001) or emotional (e.g., see Hummon, 1992; Low, 1992; Lewicka, 2014) nature of place attachment. In fact, as stated by Korpela (2012) in his review on this issue, even though cognitive and behavioral components are often included in place attachment definitions (e.g., see Jorgensen and Stedman, 2001; Scannell and Gifford, 2010), its distinctive features are represented by affects, emotions and feelings.

The construct of place attachment has recently received increasing attention, as witnessed by its theoretical developments (e.g., the tripartite model of Scannell and Gifford, 2010, the circular model of Morgan, 2010, the 5-stage model by Devine-Wright, 2009). Its relationship with a range of relevant phenomena has been verified, such as the connections with community participation (Anton and Lawrence, 2014) and with qualities of the urban residential neighborhood (Fornara et al., 2018). Less studied associations also emerged, such as relations with space appropriation (Rioux et al., 2017), acceptance of power lines (Devine-Wright, 2013), responses to natural hazard risks (Bonaiuto et al., 2016a), restorativeness (Ratcliffe and Korpela, 2016, 2018), walkability (Ferreira et al., 2016), architectural style of elderly facilities (Cerina et al., 2017), pro-environmental behaviors (Ramkissoon et al., 2013).

Among the places to which people develop attachment bonds, one of the most prominent in an individual’s life is typically the place of residence, that can be conceived at different levels, i.e., home, residential block, neighborhood, town/city level, or even broader levels (Bonaiuto et al., 2006; Bonaiuto and Alves, 2012). In this regard, residential attachment (but also identification with the place of residence) has been found to be an important antecedent of residential satisfaction (Amérigo and Aragonés, 1997; Fleury-Bahi et al., 2008; Bonaiuto et al., 2015), i.e., the experience of pleasure or gratification deriving from living in a specific place (Bonaiuto et al., 2006). The latter is considered in relation to more general patterns such as perceived quality of life, life satisfaction and subjective well-being (Di Masso et al., 2017). Marans (2003) proposed an inter-connected multi-level model where residential satisfaction is the outcome level, deriving from objective conditions (e.g., density, traffic counts, distance to nearest parks) at the first level and subjective responses (e.g., residents’ assessment of crowding, traffic, parks) at the second level. Residential attachment can be conceived as a prominent subjective response in this sense.

It is not surprising that displacement or relocation events could provoke the breakage of attachment bonds with the residential place (Fried, 1963; Manzo et al., 2008), particularly in cases of forced relocation, and this occurrence can be of particular importance for the well-being of elderly people (Cerina and Fornara, 2011). In fact, the place of residence assumes a salient meaning in the old age, for at least two reasons (Fornara and Manca, 2017): (i) elderly people usually spend most of the day in their residential environment (their home and their neighborhood; e.g., Bonaiuto et al., 2004); and (ii) the residential environment may help in providing a sense of continuity with the past (Korpela, 2012), maintaining a positive self-image (Rubinstein and Parmelee, 1992), and promoting identity, independence, and well-being (Eyles and Williams, 2008).

One important conceptual framework that has been created for understanding how, and to what extent, the residential environment plays a role in the older adults’ well-being and quality of life is the notion of Person-Environment (P-E) fit (Lawton and Nahemow, 1973; Kahana, 1982) and related models and constructs, such as the Complementary-Congruence Model of P-E fit (Carp and Carp, 1984), the P-E compatibility (Kaplan, 1983), the environmental support (Bonaiuto and Alves, 2012), and the environmental accessibility (Iwarsson and Stahl, 2003). It is to mention that also the well-known Flow theory stresses the positive consequences of a proper balance between personal skills and activity challenges (Csikszentmihalyi, 1990, 2000).

Within this perspective, a key pattern is represented by the “everyday competence” (Lawton, 1982; Willis, 1996), which refers to the individual’s ability to perform a set of activities that are considered as necessary for living in an independent way. The decline of everyday competences was found to be related to a decrease in self-esteem and life satisfaction (Kuriansky et al., 1976), and related to an increase of the use of home healthcare services (Wolinsky et al., 1983), of the risk of hospitalization and institutionalization (Branch and Jette, 1982), and of mortality (Keller and Potter, 1994). Lawton (1982) has postulated the “docility hypothesis,” which assumes that the lower the elderly person’s competence is, the higher is her/his dependence on the environment. Individual competence has also been considered as one of three fundamental psychological needs in the Self-Determination Theory (SDT; Ryan and Deci, 2000). In this sense, feelings or perceptions of competence related to an activity or domain are theorized to be important because they facilitate people’s goal attainment and provide them with a sense of satisfaction of needs from engaging in an activity where they feel effective. Competence in exerting control over one’s own environment has also been reported as one of the most important psychological resources that elderly people own (Steverink et al., 2001). In facing relocation events, environmental competence has been defined as the feeling of confidence of older individuals in their ability to adapt themselves to a non-familiar place (Cerina and Fornara, 2011).

A parallel can be suggested with perceived self-efficacy, which is known to be a crucial component of the Vested Interest Theory (VIT) based communication strategy to be adopted in order to increase inhabitants’ compliance with natural hazard risk coping (e.g., in case of flooding, De Dominicis et al., 2015): this shows that when a person is facing an increased environmental demand up to a real (even life-threatening) environmental challenge happening in her/his own place, self-efficacy reliance is one of the components favoring a proper environmental risk perception-coping relation. This kind of phenomenon is opposite to the topic addressed here, because it happens due to an increase of the environmental challenge (natural hazard affecting an urban place) in face of a constant human skill (in adulthood); on the contrary, the topic addressed by the present work has to do with a stability of the environmental challenge (a stable standard urban place) in face of a decreased human ability (in the old age). However, these two situations are psychologically equivalent from a theoretical point of view, if we frame them in terms of the Flow theory (e.g., Csikszentmihalyi, 1990, 2000), which conceives the human experience as resulting from the human skill / environmental challenge ratio as follows: apathy or boredom (low skill and low challenge), relaxation (high skill and low challenge), anxiety (low skill and high challenge), flow (high skill and high challenge). If we look at the two situations mentioned above, they are psychologically equivalent within this framework: in fact, if an adult person with stable skills has to face an overcoming challenging environmental situation (as in the natural hazard), the skill/challenge ratio becomes negatively unbalanced (in this case, due to an increase in the environmental challenge) and the resulting experience is therefore anxiety; similarly, if an older person decreases in her/his abilities to cope with a stable standard environment (as in the urban places we considered), again the skill/challenge ratio becomes negatively unbalanced (this time due to a decrease in the skill) and the resulting experience is anxiety too. Links between such a framework’s parameters balance changes and corresponding changes in environmental psychology constructs (such as place identity) have been recently demonstrated (Bonaiuto et al., 2016b). This framework, therefore, theoretically argues in favor of changes in the person’s skill/environment’s challenge balance (due to one or the other asymmetrically increasing or decreasing) and corresponding increases or decreases in the individual’s psychological states, such as anxiety.

Following this theoretical background, the central aim of the present research is to verify whether residential attachment is a possible adaptive response for elderly people, in line with the “docility hypothesis,” in order to minimize the effect of a decreasing environmental competence when coping with the demands of non-familiar environments. Diverse conceptual dichotomies have been proposed to describe the coping strategies that the elderly use to respond to environmental demands, i.e., proactivity vs. reactivity (Lawton, 1989), assimilation vs. accommodation (Brandtstädter and Renner, 1990), adaptation to the environment vs. self-adaptation (Slangen-de Kort et al., 1998). In these dichotomies, the first pole refers to the “active” tendency to adjust life circumstances to personal preferences, whereas the second pole concerns to the “passive” tendency of adjusting personal preferences to situational constraints.

In this paper, environmental competence is conceived in terms of spatial ability and related patterns, such as spatial self-efficacy, spatial anxiety, and spatial orientation. Spatial ability is the cognitive skill needed to encode, maintain and process visuo-spatial information (Lohman, 1988). It is distinguished into two types of skills, partially overlapping (Hegarty et al., 2006): small-scale spatial skills, which are characterized by spatial tasks that require the mental manipulation or transformation of shapes or objects, and large-scale spatial skills characterized by tasks that require physical or imagined movement through spatial environments. Spatial ability is expected to decline to some degree with aging (Devlin, 2001). Proximal dimensions of spatial ability are: (a) spatial self-efficacy, based on Bandura’s Self-Efficacy Theory (Bandura, 1997), which concerns how effectively people feel they deal with typical tasks that require spatial skills (Pazzaglia et al., 2018); (b) spatial anxiety (Lawton, 1994), concerning how anxious people feel about tasks that require spatial skills; and (c) spatial orientation, that is the ability to ascertain our own position in relation to the surrounding environment (Mitolo et al., 2015).

Therefore, our main research question is whether place attachment to one’s own residential environment, at the level of the residential neighborhood, works as a coping strategy of reactivity (or accommodation or self-adaptation) to diminished (or anyway lower) spatial ability skills when interacting with unfamiliar places in the old age. Our goal is in line with the process-oriented direction suggested by Lewicka (2011b) in her influential review of place attachment research. To the best of our knowledge, the present study is the first attempt to examine the relationship between space experience patterns and the environmental-psychological pattern of place attachment.

## Objective and Hypotheses

The objective of the study is to verify the possible buffer role of place attachment between decreased spatial competence and residential satisfaction in older people. In particular, the elder’s positive attitude toward way-finding tasks, which emerged as associated to orientation ability (Pazzaglia et al., 2017), is postulated to depend on their own spatial self-efficacy, which is supposed to regulate the level of anxiety triggered by environmental demands. A higher attachment to one’s own residential environment would play the role of a “reactive” response of “spatial closure” to a low attitude toward way-finding tasks, which specifically characterizes the environmental demands of non-familiar places. Given that residential satisfaction - which is related to more general patterns such as life satisfaction and quality of life (Di Masso et al., 2017) - has shown to be positively predicted by residential attachment (Amérigo and Aragonés, 1997), the latter dimension would play a compensative role between reduction of skills and well-being in the aged person.

Our hypotheses were thus organized around a theoretical sequential path including, respectively: (i) the relationships among the spatial competence variables, (ii) the relationships of these with the residential attachment and, finally, (iii) the relationship between residential attachment and residential satisfaction. Specifically, we expected that (H1) a low (vs. high) spatial self-efficacy could trigger a high (vs. low) spatial anxiety, which in turn (H2) promotes a negative (vs. positive) attitude toward way-finding tasks in unfamiliar environments. This would elicit (H3) a higher (vs. lower) attachment to one’s own residential place. Finally, we expected that (H4) the higher the residential attachment, the higher the residential satisfaction. The tested model included possible connections between non-contiguous dimensions in the sequential path, except for the final dimension, i.e., residential satisfaction, which was expected to be predicted only by residential attachment. Participants’ age and length of residence in the neighborhood were also taken into account, given their proven positive relationship with place attachment (Bonaiuto et al., 1999), especially concerning length of residence (Hernandez et al., 2007; Tabernero et al., 2010; Manzo and Devine-Wright(eds), 2014), whereas mixed evidence emerged for age (Lewicka, 2011a,b).

## Materials and Methods

### Sample and Procedure

The sample consists of 264 participants (65.2% Females, 34.8% Males) of Italian nationality, aged between 65 and 96 years (*M* = 74.34; *SD* = 6.64). The average years of residence in the municipality is 44.72 (from 1 to 87; *SD* = 21.30). The majority of the participants are high school graduates (33.5%), followed by those with a primary school education (25.0%), a middle school education (24.6%), and those with a degree (16.9%). Data were collected in diverse neighborhoods of different Italian cities during the years 2017 and 2018 by various trained interviewers, i.e., post-graduated psychologists and architects enrolled in a Master at the University of Padua, and Psychology students from the Universities of Padua and Cagliari. The interviewers were told to contact potential participants starting from their connections, and then to find further participants by following a snowball sampling procedure. Other participants were recruited in parks, urban gardens, and senior leisure centers. All participants were self-sufficient and lived in their homes alone or with their relatives. Before administrating the study questionnaire, interviewers verified that participants were able to respond lucidly to the questionnaire items.

This study was carried out in accordance with the recommendations of the Ethics Committee for Psychological Research (CERP; Comitato Etico per la Ricerca Psicologica; *http://ethos.psy.unipd.it/*) of the University of Padua, which approved the research protocol. All participants gave their written informed consent in accordance with the Declaration of Helsinki.

After obtaining the participant’s informed consent, the interviewer had the task to administer a paper-format questionnaire and to follow its compilation, particularly in those cases where the participant needed support. The average response time for the questionnaire compilation was approximately 40 min.

### Measures

The study questionnaire included the following measures.

#### Perceived Spatial Self-Efficacy

This variable was measured through the Spatial Self-Efficacy Scale (Pazzaglia et al., 2018), which included 4 items (α = 0.91). Participants were asked to indicate their degree of perceived self-efficacy on a 5-point Likert-type scale (from 1 = “not at all” to 5 = “very much”) in specific situations, e.g., “To go alone to visit someone who lives in a place you do not know well,” and “To get out of a mall and decide which direction to take to get home.”

#### Perceived Spatial Anxiety

This variable was measured through the Spatial Anxiety Scale (Mitolo et al., 2015), adapted from Lawton (1994), which included 6 items (α = 0.89). Participants were asked to indicate their degree of perceived anxiety on a 5-point Likert-type scale (from 1 = “not at all” to 5 = “very much”) in specific situations, most of them equal to those used for measuring Perceived spatial self-efficacy.

#### Attitude Toward Wayfinding

This variable was measured through the Scale of Attitudes toward wayfinding tasks (Pazzaglia et al., 2017), which included 4 items (α = 0.75), e.g., “I’ve always enjoyed exploring different places that I do not know well to discover new paths and different places,” and “Before leaving for a trip and/or a holiday I’ve always enjoyed finding the route on the map and where the places to visit are.” Participants were invited to evaluate each statement through a 5-point Likert-type scale (from 1 = “not at all” to 5 = “very much”).

#### Residential Attachment

This variable was measured through the short version of the Neighborhood Attachment Scale (Fornara et al., 2010). From the original 4 items, 3 items were retained (α = 0.87), i.e., “This neighborhood is part of me,” “This is the ideal neighborhood for me,” and “It would be very hard for me to leave this neighborhood.” For each statement, Participants were invited to express their degree of agreement or disagreement through a 7-point Likert-type scale (from 0 = “totally disagree” to 6 = “totally agree”).

#### Residential Satisfaction

This variable was measured at the neighborhood level through the Residential Satisfaction Scale (Bonaiuto et al., 2015), adapted from Fornara et al. (2006), that includes 3 items (α = 0.88), i.e., “Overall, how satisfied are you to live in this neighborhood?,” “Would you recommend this neighborhood to friends or acquaintances who are looking for a house?,” and “Do you intend to live in this neighborhood for long time?” The participants were invited to evaluate each statement through a 7-point Likert-type scale (from 0 = “not at all” to 6 = “completely”).

### Data Analysis

**FIGURE 1:**
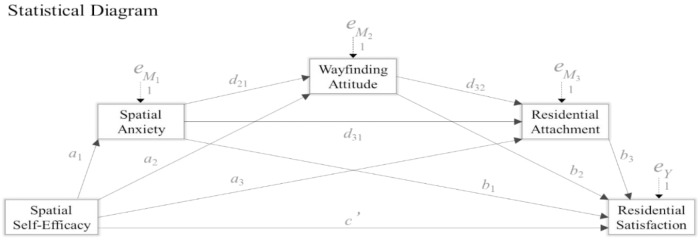
Statistical diagram of the serial multiple mediation model including the direct and indirect effects of spatial self-efficacy on residential satisfaction.

Data were analyzed with SPSS 23.0. Descriptive statistics, reliability analysis, and inter-correlation matrix were computed for the study variables. A serial mediation model (multiple-step multiple mediation; Hayes et al., 2010) was tested using PROCESS version 3.1 (see SPSS, Hayes, 2018), with 5.000 sample bootstrapping technique and 95% confidence intervals. A statistical diagram of the selected model, i.e., model 6, is presented in Figure 1. In order to estimate the coefficients in the model, and to determine the direct and indirect effects of spatial self-efficacy on residential satisfaction, an ordinary-least-squares path analysis was run, controlling for age and length of residence in the neighborhood.

## Results

Table 1 reports means, standard deviations and Pearson’s *r* bivariate correlations of the study variables. It is to notice that the average means of residential attachment and satisfaction are rather high, whereas spatial self-efficacy and way-finding attitude are slightly above the mean point of the scale, and spatial anxiety is below the mean point of the scale.

**Table 1:** Descriptive statistics and correlation coefficients.

Variable	*M*	*SD*	1	2	3	4	5
(1) Spatial self-efficacy	3.10	0.94	(0.91)				
(2) Spatial anxiety	2.10	0.83	–0.397^∗∗∗^	(0.89)			
(3) Wayfinding attitude	3.24	0.95	0.455^∗∗∗^	–0.417^∗∗∗^	(0.75)		
(4) Residential attachment	4.52	1.46	–0.016	0.080	–0.232^∗∗∗^	(0.87)	
(5) Residential satisfaction	4.94	1.18	0.016	0.028	–0.110	0.717^∗∗∗^	(0.88)


*Reliability coefficients (Cronbach’s Alphas) are presented in parentheses on the diagonal. ^∗∗∗^*p* < 0.001.*

**FIGURE 2:**
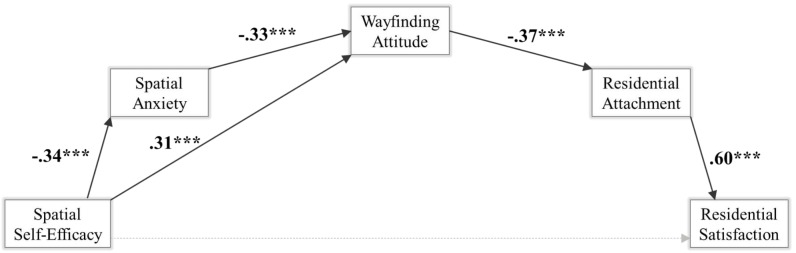
Serial multiple mediation model with spatial anxiety, spatial attitude, and residential attachment as mediators of spatial self-efficacy effects on residential satisfaction. ^∗∗∗^*p* < 0.001.

Figure 2 and Table 2 present the overall outcome and the detailed parameters of the tested multiple-step mediation model.

**Table 2:** Regression coefficients, standard errors, and model summary information of the tested serial multiple mediation model.

		Consequent														
		*M*_1_ (Spatial Anxiety)				*M*_2_ (Wayfinding Attitude)				*M*_3_ (Residential Attachment)				*Y* (Residential Satisfaction)		
								
Antecedent		Coefficient	*SE*	*p*		Coefficient	*SE*	*p*		Coefficient	*SE*	*p*		Coefficient	*SE*	*p*
*X* (Spatial Self-Efficacy)	*a*_1_	–0.341	0.051	< 0.001	*a*_2_	0.307	0.057	< 0.001	*a*_3_	0.200	0.108	*ns*	*c′*	–0.006	0.063	*ns*
*M*_1_ (Spatial Anxiety)		–	–	–	*d*_21_	–0.326	0.065	< 0.001	*d*_31_	0.028	0.121	*Ns*	*b*_1_	0.012	0.071	*ns*
*M*_2_ (Wayfinding Attitude)		–	–	–		–	–	–	* d*_32_	–0.372	0.111	< 0.001	*b*_2_	0.076	0.066	*ns*
*M*_3_ (Residential Attachment)		–	–	–		–	–	–		–	–	–	*b*_3_	0.595	0.036	< 0.001
Covariates																
Years of residence		–0.004	0.002	*ns*		–0.005	0.002	< 0.05		0.003	0.004	*ns*		0.005	0.003	*ns*
Age		0.010	0.007	*ns*		–0.021	0.008	< 0.01		0.029	0.014	< 0.05		–0.012	0.008	*ns*
Constant	j_M1_	2.617	0.587	< 0.001	j_M2_	4.794	0.636	< 0.001	j_M3_	2.745	1.256	< 0.05	j_Y_	2.668	0.740	< 0.001
		*R*^2^ = 0.173				*R*^2^ = 0.317				*R*^2^ = 0.084				*R*^2^ = 0.527		
		*F*(3,260) = 18.140 *p* < 0.001				*F*(4,259) = 29.997 *p* < 0.001				*F*(5,258) = 4.721 *p* < 0.001				*F*(6,257) = 47.666 *p* < 0.001		


In the first step of the model [*F*_3,260_ = 18.14, *p* < 0.001, *R*^2^ = 0.17], perceived spatial self-efficacy significantly negatively predicts perceived spatial anxiety (*b* = -0.34, *t*_260_ = -6.74, *p* < 0.001), in line with H1.

In the second step of the model [*F*_4,259_ = 30.00, *p* < 0.001, *R*^2^ = 0.32], way-finding attitude is significantly predicted by both perceived spatial anxiety (negatively, *b* = -0.33; *t*_259_ = -5.04, *p* < 0.001), thus confirming H2, and spatial self-efficacy (positively, *b* = 0.31; *t*_259_ = 5.36, *p* < 0.001). Both the covariates – age (*b* = -0.02, *t*_259_ = -2.77, *p* < 0.01) and length of residence (*b* = -0.01, *t*_259_ = -2.28, *p* < 0.05) – show a significant negative relationship with way-finding attitude.

In the third step of the model [*F*_5,258_ = 4.72, *p* < 0.001, *R*^2^ = 0.08], residential attachment is significantly negatively predicted by way-finding attitude (*b* = -0.37; *t*_258_ = -3.35, *p* < 0.001), thus corroborating H3; whereas perceived spatial self-efficacy (*b* = 0.20; *t*_258_ = 1.86, *p* = *ns*) and perceived spatial anxiety (*b* = 0.03; *t*_258_ = 0.23, *p* = *ns*) were both non-significant. The covariate age shows a significant positive relationship (*b* = 0.03, *t*_258_ = 2.09, *p* < 0.05) with residential attachment.

In the last step of the model [*F*_6,267_ = 47.67, *p* < 0.001, *R*^2^ = 0.52], the final outcome variable, i.e., residential satisfaction, is significantly positively predicted by residential attachment (*b* = 0.60; *t*_267_ = 16.39, *p* < 0.001), in line with H4, whereas perceived spatial self-efficacy (*b* = -0.01; *t*_267_ = -0.10, *p* = *ns*), perceived spatial anxiety (*b* = 0.01; *t*_267_ = 0.16, *p* = *ns* ), and spatial attitude (*b* = 0.08; *t*_267_ = 1.15, *p* = *ns*) were, as expected, non-significant.

The total effect [*F*_3,260_ = 1.82, *p* = *ns*, *R*^2^ = 0.02] of spatial self-efficacy (*b* = 0.04, *t*_260_ = 0.54, *p* = *ns*) on residential satisfaction was non-significant, nevertheless this result does not undermine the mediational path. In fact, as stated by Hayes (2018), p. 117), “the size of the total effect does not constrain or determine the size of the indirect effect […] An indirect effect can be different from zero even when the total effect is not.”

Only the indirect effect of *X* on *Y* through *M*_1_, *M*_2_, and *M*_3_ in serial = *a*_1_ *d*_21_ *d*_32_ *b*_3_ [β = -0.02, SE = 0.01 (CI = -0.04, -0.01)], and the indirect effect of *X* on *Y* through *M*_2_, and *M*_3_ in serial = *a*_2_ *d*_32_ *b*_3_ were significant [β = -0.05, SE = 0.02 (CI = -0.11, -0.02)].

Coefficients for the serial mediation model are presented in Figure 2.

## Discussion and Conclusion

The study findings provide a first evidence to the buffer role of residential attachment, as an intermediate dimension between negative antecedents (i.e., low spatial abilities) and a positive outcome (i.e., residential satisfaction). In fact, the hypothesized sequential path connecting the spatial competence dimensions, via residential attachment, to residential satisfaction is confirmed. Specifically, a low spatial self-efficacy is associated with a high spatial anxiety (H1), consistently with the Flow theory predictions (Csikszentmihalyi, 1990, 2000). This in turn elicits a more negative attitude toward way-finding tasks (H2). There is also a direct positive link between spatial self-efficacy and attitude toward way-finding tasks that is the only connection between non-proximal variables in the sequential path. This link is not surprising and gives an empirical response to those who claimed that it might be useful to verify “whether and to what extent higher levels of anxiety and lower self-efficacy ratings may negatively influence an individual’s orientation skills” (Mitolo et al., 2015, p. 174).

A negative attitude toward way-finding tasks, which reflects low spatial orientation skills (Pazzaglia et al., 2017), is related to a higher residential attachment (H3), which is a higher (vs. lower) attachment to one’s own residential place. This is in line with the “docility hypothesis” (Lawton, 1982), since the higher attachment to one’s own residential environment can be seen as an example of place dependency or closure, in response to the depletion of spatial competence resources. Within this framework, residential attachment should mirror a “reactivity” (Lawton, 1989), “accommodation” (Brandtstädter and Renner, 1990), and “self-adaptation” (Slangen-de Kort et al., 1998) strategy used by older adults for compensating successfully the reduction of their spatial abilities when coping with unfamiliar environments, and therefore overcoming the consequential anxiety. Finally, the role of residential attachment is confirmed (H4) as a strong predictor of residential satisfaction (Amérigo and Aragonés, 1997; Fleury-Bahi et al., 2008; Bonaiuto et al., 2015).

In sum, attachment to the residential environment seems to resemble a “passive” adaptive coping strategy to tackle the decline of spatial abilities that may occur in old age, even though further empirical evidence is needed. This process could be evaluated through the lens of Bronfenbrenner (1979) systemic view and the conception of people’s responses as part of a broader multi-place system of activities, which may also link together a person’s pragmatic system with her/his own perception and evaluation of the sub-places where s/he is living in the city (Bonaiuto et al., 2004). Within this framework, each place is a system of subplaces which relate each other according to the criteria of “inclusion versus exclusion” and “nearness versus farness.” Hence, the different subplaces are more or less connected with reference to users’ goals, activities, representations, and opportunities (Bonnes and Secchiaroli, 1995). Thus, it is important to detect the patterns of the activities and their “locatedness” (Dixon, 2001) in order to understand the responses of adjustment or maladjustment (Bonaiuto and Alves, 2012), which are related to the P-E fit (Lawton and Nahemow, 1973; Kahana, 1982). In this case, the elders’ high attachment to their residential environment could be a “closure” response which is consistent with the findings of a previous study, where older adults showed a low urban mobility and substantial confinement in their residential neighborhood (Bonaiuto et al., 2004). On the other hand, familiar environments should play a positive role of promoting functional and social competence in the elderly (Cerina et al., 2017).

Further research is needed to confirm the emerged relationships with other samples and in other contexts. In particular, one limitation of the study is related to the verification of a temporal sequence through a cross-sectional design. Longitudinal research is thus needed in order to corroborate the sequential path here tested. A second limitation concerns the variety of urban features which characterize the places where participants live, though we conceive the diversity of such features as a random variable which should not have affected the relationships between spatial abilities and residential attachment. A third limitation is the lack of a specific measure of the mental state of respondents, thus we had to rely on the judgment of the interviewers.

Another important question to address concerns whether or not this pattern of relationships characterizes only older human beings. What about other ages? Could this confinement response, reflected by a high residential attachment, emerge also in other age ranges? Moreover, such pattern could possibly find some interesting parallels outside the human species, as it refers to very basic survival strategies which may be relevant for other animal species in their ecosystems too.

## Ethics Statement

This study was carried out in accordance with the recommendations of the Ethical Committee of the Inter-departmental Psychology Area of the University of Padua, which approved the research protocol. All participants gave their written informed consent in accordance with the Declaration of Helsinki.

## Author Contributions

FF, MB, and FP were involved in the planning of the study. FF wrote “Introduction,” “Objective and Hypotheses,” and “Discussion and Conclusion” sections of the manuscript, and revised the “Materials and Methods,” and “Results” sections. AL performed the data analysis and wrote the “Materials and Methods,” and “Results” sections. MB and FP revised the manuscript.

## Conflict of Interest Statement

The authors declare that the research was conducted in the absence of any commercial or financial relationships that could be construed as a potential conflict of interest.
